# Clinical-epidemiological profile and factors associated with viral non-suppression in patients living with HIV/AIDS assisted at the Integrated Treatment Center at the Maputo Military Hospital (CITRA/MMH), 2019 to 2020

**DOI:** 10.1186/s12879-024-09616-2

**Published:** 2024-07-20

**Authors:** Eduardo Mangue Chicanequisso, Cynthia Sema Baltazar, Jahit Sacarlal

**Affiliations:** 1 Mozambique Field Epidemiology and Laboratory Training Program, National Institute of Health, P.O. Box 264, Maputo, Mozambique; 2 Militar Health Department, General Staff of the Mozambique Armed Defense Forces, Maputo, Mozambique; 3 National Institute of Health, Maputo, Mozambique; 4 Microbiology Department, Faculty of Medicine, Eduardo Mondlane University, Maputo, Mozambique

**Keywords:** Viral non-suppression, Viral load, HIV, ART, Mozambique, Epidemiologic Factors

## Abstract

**Background:**

HIV remains a critical global public health challenge. In 2022, it was estimated that approximately 39.0 million people worldwide were living with HIV, and of these, around 29.8 million were receiving antiretroviral therapy (ART). The objective was to evaluate the clinical and epidemiological profile and factors associated with viral load (VL) non-suppression in people living with HIV/AIDS at the Maputo Military Hospital (CITRA/MMH).

**Methods:**

A retrospective cross-sectional analytical study was conducted on 9105 people aged 15 years and over. We use secondary data from participants on ART for at least 2 years being followed up between the years 2019–2020 at CITRA/MMH. Those recently enrolled (on ART < 1 year) were excluded and data analysis was performed using STATA version 16. Pearson's chi-square test and logistic regression were used for statistical modeling of viral non-suppression with a 95%/CI confidence interval and *p* < 0.05.

**Results:**

Among a total of 9105 HIV participants included, 52.8% (*n* = 4808) were female and 13.6% (*n* = 1235) were military personnel. The average age was 47.9 years (standard deviation ± 12.1), with the most prevalent age group being individuals aged between 25 and 59, totalizing 7,297 (80.2%) participants. Only 5395 (100%) participants had VL results. Among these, 23.1% (*n* = 1247) had a result VL non-suppressed. Single marital status (Adjusted Odds Ratio [AOR] = 4.8, 95%CI: 3.93–5.76, *p* < 0.001), with active tuberculosis (AOR = 4.6, 95%CI: 3.15–6.63, *p* < 0.001) and current ART regimen in categories TDF + 3TC + EFV (AOR = 12.7, 95%CI: 9.74–16.63, *p* < 0.001), AZT + 3TC + NVP (AOR = 21.8, 95% CI: 14.13–33.59, *p* < 0.001) and “other” regimens (AOR = 25.8, 95%CI: 18.58–35.80, *p* < 0.001), when compared to the TDF + 3TC + DTG regime, were statistically significant for viral non- suppression.

**Conclusion:**

The study highlights the crucial role of ART adherence and ongoing monitoring to achieve viral suppression, particularly among adults aged 25 to 59. It underscores the need for transitioning eligible individuals to DTG-based regimens and addressing the implications of single marital status and comorbid conditions like active tuberculosis. The study emphasizes the importance of ARV adherence and continuous monitoring to meet the UNAIDS 95–95-95 targets and improve clinical outcomes for people living with HIV/AIDS.

## Background

HIV remains a critical public health challenge worldwide. As of 2022, approximately 39 million people globally were living with HIV, with Eastern and Southern Africa being the most impacted regions. These areas account for 53.3% (20.8 million) of all people living with HIV/AIDS (PLHIV) [1].

Within Southern Africa, Mozambique is one of the most affected countries, with about 2.5 million PLHIV. Estimates for 2022 suggest roughly 97,000 new HIV infections and 48,000 HIV-related deaths in the country [2]. The most recent data from the National Survey on the Impact of HIV and AIDS in Mozambique (INSIDA 2021) indicated an HIV prevalence of 12.5%, which corresponds to approximately 2,097,000 PLHIV adults in Mozambique. HIV prevalence was higher among women (15.0%) than men (9.5%). Among those on antiretroviral therapy (ART), the prevalence of viral load (VL) suppression is 89.4%, with a slightly higher rate in women (90.4%) than in men (87.6%) [3].

The impact of HIV/AIDS extends significantly into military settings, where it compromises not only the operational capabilities but also the overall wellbeing of service members. Globally, it's observed that the presence of HIV/AIDS among military personnel and similar uniformed groups detrimentally affects their preparedness and operational capacity. Additionally, it restricts their professional endeavors and leads to a deterioration in both the physical health and emotional wellbeing of not only the affected individuals but also their families [4].

The circumstances experienced within the military environment are very challenging due to high mobility and long periods away from home. These circumstances increase the possibility of casual sexual relationships, which can put military personnel at an increased risk of contracting sexually transmitted infections (STI’s), including HIV. Military personnel are at a high risk of occupational injuries due to their duties, which increases the likelihood of acquiring HIV through contact with infected blood or open wounds [5]. According to data from the HIV Program of the Mozambican Armed Forces (FADM), only 46.5% (6,483 out of 13,953) had their VL tested. Among those tested, 86.3% (5,593/6,483) achieved viral suppression [6].

Ongoing and systematic care for PLHIV, including consistent access to ARVs to maintain viral suppression, is fundamental for prevention and control of HIV. This approach is vital for allowing immune reconstitution, minimizing the emergence of drug resistance, reducing HIV-related morbidity and mortality, and preventing the transmission of drug-resistant HIV mutations [7, 8]. Monitoring individuals on ART is critical on HIV response. It not only ensures the effectiveness of treatment but also helps identify adherence issues and determine when changes in ART regimens are needed in cases of treatment failure [9]. The World Health Organization (WHO) recommends conducting VL tests within six months of starting ART and annually thereafter [10].

Despite the importance of regular ARV administration, it does not always guarantee viral suppression. Furthermore, therapeutic failure can occur even with consistent ARV usage, owing to a variety of individual and population-specific factors, as well as systemic healthcare challenges. Hence, context-specific data is crucial to implement effective measures for viral suppression and to enhance the quality of life for PLHIV [11].

This study aims to evaluate the clinical-epidemiological profile and factors associated with viral non-suppression in PLHIV at the Integrated Treatment Centre at Maputo Military Hospital (CITRA/HMM), Mozambique.

## Methods

### Study design and setting

A hospital-based cross-sectional analytical study was conducted. Secondary data were retrospectively collected from an existing database at the CITRA/MMH. CITRA is a center dedicated to providing medical care, particularly to PLHIV within the military community, including service members and their dependents.

The Maputo Military Hospital has a Health Care and Testing Unit, which serves as a sit for HIV testing. This unit is accessible to everyone, regardless of military affiliation, ensuring broad community outreach. Upon identification of HIV-positive individuals, all military personnel and their dependents are referred to the CITRA. CITRA then initiates the comprehensive process of follow-up, care, and treatment for these patients. Functioning as a specialized unit attached to the Hospital, CITRA is dedicated to providing tailored care for HIV-positive individuals, including both civilians and military personnel.

### Data collection

The study included PLHIV who are enrolled in the HIV-related care and assistance service and have had at least one visit between January 2019 and December 2020, are aged 15 years or older, and have initiated ART. Data from pregnant patients were excluded from the study due to inherent clinical management specificities, as well as HIV-positive patients with less than two years of follow-up.

### Data analysis

The data was extracted from the OpenMRS platform, which is used for entering and storing patient data at the CITRA/HMM HIV service. In instances where certain variables were missing from the electronic database, clinical files were reviewed to supplement and complete the dataset. For participants who had multiple follow-ups within the study period, only data from the most recent consultation was considered. This data was initially entered into the database derived from the *OpenMRS* platform in an Excel format. Subsequently, the data was transferred from the *Microsoft Office Excel* spreadsheet, and then underwent a process of cleaning, coding, and analysis.

The statistical analysis was performed using *STATA* version 16. This included both descriptive (frequencies, means, and standard deviation) and inferential statistical techniques, employing 95% confidence intervals (CI) and hypothesis tests at a 5% significance level. For inferential statistics, logistic regression was utilized, focusing on the dichotomous dependent variable of viral suppression status, based on the most recent VL laboratory test. Viral suppression was defined based on the national guidelines, as a patient who has a VL < 1000 copies/ml and non-suppressed VL was defined as VL >  = 1,000 copies/ml. The analysis focused on the most recent VL results available for each participant within the study period of 2019–2020. This dichotomous variable was derived from the results of the last VL test. All variables that showed statistical significance in the bivariate analysis were included in the final multivariate logistic regression model.

### Ethical consideration

This research was approved by the Institutional Health Bioethics Committee of the Faculty of Medicine/Central Hospital of Maputo (CIBS FM&HCM), under reference CIBS FM& HCM/050/2021. Administrative approval was obtained from the General Directorate of the HMM, which in turn issued a credential in favor of the principal investigator, granting permission to access and use the data. The data came from a secondary database of PLHIV for follow-up and treatment. The data came from a secondary database of PLHIV for follow-up and treatment. The data was not accessible to third parties other than the study team.

## Results

### Sociodemographic and clinical characteristics of patients on ART at CITRA

From the database analyzed, a total of 9,105 participants met the inclusion criteria. Over half (52.8%) were female, with a median age of 47 years (IQR: 40–56 years). The 45–54 and 55 + age groups were the most prevalent, accounting for 29.8% and 28.5% of participants, respectively. Marital union/married was the majority, with 81.7% reporting this status.

The majority (approximately 81.5%) were civilians receiving care, with a significant portion (62.0%) having been followed for 10 or more years (Table 1). This extended follow-up period suggests a well-established program.

**Table 1:** Sociodemographic and clinical data patients on ART at CITRA with a Minimum Follow-Up Period of 2 Years, 2019–2020

Variable	Frequency (*N* = 9105)	Percentage
**Age in years**		
15–24	268	2.9
25–34	887	9.7
35–44	2643	29.0
45–54	2709	29.8
55 +	2598	28.5
*Median age*	47 (IQR^a^ 40–56)	
**Female gender**	4808	52.8
**Marital status**		
Marital union/married	7435	81.7
Single/divorced	1670	18.3
Provenience’s province (***N*** = 2844)		
City of Maputo	1707	60.0
Maputo Province	1101	38.7
Other	36	1.3
Patient type (***N*** = 8658)		
Military	1235	14.3
Civil	7423	85.7
**Years of follow-up**		
2–5	1894	20.8
6–9	1562	17.2
10 +	5649	62.0
**Patient outcome**		
Active	8447	92.8
Transferred	334	3.7
Abandonment	168	1.8
Death	156	1.7
WHO stage in the baseline (***N*** = 8196)		
I	2833	34.6
II	2021	24.7
III	2717	33.1
IV	625	7.6
Most recent ART regimen (***N*** = 9098)		
TDF + 3TC + DTG	4613	50.7
TDF + 3TC + EFV	1409	15.5
AZT + 3TC + NVP	1110	12.2
Other^c^	1966	21.6
No suppressed viral load (≥ 1000 copies/mL) (***N*** = 5395)	1247	23.1
**Tuberculosis positive result**	230	2.5
**Have Kaposi's sarcoma**	284	3.1

^*a*^*IQR-Interquartile ranges,*

^*b*^*TDF-tenoforvirdisoproxifumarate, 3TC-lamivudine, DTG- dolutegravir, EFV-enfaverence, AZT-azidothymidine, NVP-nevirapine*

^*c*^*Others included a variety of combinations not classified under the primary categories listed, and not specified on the database*

VL data was available for 59.3% of participants. Among these, a non-suppressed VL (≥ 1000 copies/mL) was present in 23.1% (*n* = 1,247). It's important to note that 625 (7.6%) initiated ART at a later stage (WHO stage 4), which could contribute to the non-suppressed vl prevalence. The most common first-line ART regimen was TDF + 3TC + DTG (50.5%). Table 1 also highlights the presence of comorbidities, with 2.5% (230/9105) having active tuberculosis and 3.1% (284/9105) diagnosed with Kaposi's sarcoma at their last visit.

### *Bivariate analysis of demography and clinical factors associated with VL suppression (*< *1000 copies) among military and civilian patients attending CITRA*

Table 2 presents the relationship between patient type (civilian or military) and various variables of interest. Regarding marital status, the majority of civilians were in a marital union, with 83.8% (6222/7423) compared to 77.3% (995/1235) of military personnel. The most common ART regimen was TDF + 3TC + DTG, used by 52.4% of participants (4537/4492), with a higher prevalence among civilians than military personnel.

A significant difference in viral load (VL) suppression was observed between military personnel and civilians. Military patients had a higher proportion of non-suppression, with 30.1% (206/685) exhibiting VL non-suppression compared to 20.0% (900/4492) of civilians. This difference in VL suppression rates was statistically significant (*p* < 0.001), indicating a notable disparity in ART service effectiveness between the two groups.

The differences between military personnel and civilians were statistically significant (*p* < 0.05) for other variables as well, including the time of follow-up, WHO stage, presence or absence of tuberculosis or Kaposi's sarcoma, and treatment outcomes. These values are detailed in Table 2.

**Table 2:** Comparison of proportions between civilian and military patients on ART at the CITRA/HMM for at least 2 years, 2019–2021 (*n* = 8658)

**Variables**	**Categories**	**Patient Profile**		**Total**	**P**
		**Military**	**Civil**		
**Sex**	Masculine	583 (47.2)	3509 (47.3)	4092 (47.3)	0.966
	Feminine	652 (52.8)	3914 (52.7)	4566 (52.7)	
**Age range**	15–24	26 (2.1)	230 (3.1)	256 (3)	0.11
	25–59	986 (79.8)	5941 (80)	6927 (80)	
	60 +	223 (18.1)	1252 (16.9)	1475 (17)	
**Marital status**	Marital union	955 (77.3)	6222 (83.8)	7177 (82.9)	< 0.001*
	Single	280 (22.7)	1201 (16.2)	1481 (17.1)	
Province of residence (***N*** = 2711)	City Maputo	222 (50.0)	1398 (59.9)	1620 (59.8)	0.87
	Maputo province	150 (39.0)	908 (38.9)	1058 (39.0)	
	Other	4 (1.1)	29 (1.2)	33 (1.2)	
**ART regimen**^**a**^	TDF + 3TC + DTG	558 (45.2)	3979 (53.6)	4537 (52.4)	< 0.001*
	TDF + 3TC + EFV	299 (24.2)	1075 (14.5)	1374 (15.9)	
	AZT + 3TC + NVP	171 (13.8)	871 (11.7)	1042 (12)	
	Other^b^	207 (16.8)	1498 (20.2)	1705 (19.7)	
**Follow-up time (years)**	2–5	137 (11.1)	1578 (21.3)	1715 (19.8)	< 0.001*
	6–9	154 (12.5)	1329 (17.9)	1483 (17.1)	
	10 +	944 (76.4)	4516 (60.8)	5460 (63.1)	
VL suppression (***n*** = 5177)	Suppressed	479 (69,9)	3592 (80,0)	4071 (78,6)	< 0,001*
	Non-suppressed	206 (30,1)	900 (20,0)	1106 (21,4)	
Baseline WHO stage (***N*** = 7841)	I	320 (28.2)	2358 (35.2)	2678 (34.2)	0.530
	II	267 (23.5)	1677 (25)	1944 (24.8)	
	III	438 (38.6)	2178 (32.5)	2616 (33.4)	
	IV	111 (9.8)	492 (7.3)	603 (7.7)	
**Tuberculosis**	No	1190 (96.4)	7242 (97.6)	8432 (97.4)	0.014*
	Yes	45 (3.6)	181 (2.4)	226 (2.6)	
**Kaposi's sarcoma**	Yes	1180 (95.6)	7199 (97)	8379 (96.8)	0.008*
	No	55 (4.5)	224 (3)	279 (3.2)	
**Outcome**	Active	1076 (87.1)	6949 (93.6)	8025 (92.7)	< 0.001*
	Transferred	79 (6.4)	246 (3.3)	325 (3.8)	
	Abandonment	47 (3.8)	111 (1.5)	158 (1.8)	
	Death	33 (2.7)	117 (1.6)	150 (1.7)	
**Total**		**1235 (14.3)**	**7423 (85.7)**	**8658 (100)**	

^*a*^*ART-antiretroviral treatment, TDF-tenoforvirdisoproxifumarate, 3TC-lamivudine, DTG- dolutegravir, EFV-enfaverence, AZT-azidothymidine, NVP-nevirapine,*

^*b*^*Others included a variety of combinations not classified under the primary categories listed, and not specified on the database *

*significant at P-value of ≤0.05

The differences between military personnel and civilians were statistically significant (*p* < 0.05). Likewise, Time of follow-up, WHO stage, presence or absence of TB or Kaposi's Sarcoma and Outcome, with the respective values in Table 2.

### Factors associated with viral non-suppression

The bivariate logistic regression analysis showed that factors such as living in a marital union, shorter follow-up time (2–5 years), being in the military, WHO stage 1, ART regimen (TDF + 3TC + EFV; AZT + 3TC + NVP and others compared to DTG), being diagnosed with TB and Kaposi's Sarcoma, outcome of each case, where those who abandoned treatment and deaths were associated with no viral suppression (see Table 3).

**Table 3:** Association of variables with viral non-suppression among adult patients on ART at HMM/CITRA for at least 2 years (*n* = 5395)

Variables	Categories	Viral suppressed status			Odds Ratio (OR)			
		**Suppressed**	**Non-suppressed**	**Total**	**Crude**		**Adjusted**	
		***n ***(%)	***n*** (%)	***n*** (%)	**OR (95%CI)**	***P***	**AOR (95%CI)**	***P***
Sex	Masculine	1997 (48.1)	598 (48.0)	2595 (48.1)	Ref			
	Feminine	2151 (51.9)	649 (52.0)	2800 (51.9)	1.0 (0.87–1.13)	0.91		
Age range	15–24	108 (2.6)	28 (2.2)	136 (2.5)	Ref			
	25–59	3309 (79.8)	1015 (81.4)	4324 (80.1)	1.2 (0.79–1.08)	0.75		
	60 +	731 (17.6)	204 (16.4)	935 (17.3)	1.1 (0.69–1.67)	0.43		
Marital status	Marital union	3429 (82.7)	423 (33.9)	3852 (71.4)	Ref			
	Single	719 (17.3)	824 (66.1)	1543 (28.6)	9.3 (8.06–10.71)	< 0.001	4.8 (3.93–5.76)	< 0.001
Province	SI	2504 (60.4)	882 (70.7)	3386 (62.8)				
	City Maputo	1025 (24.7)	214 (17.2)	1239 (23.0)	Ref			
	P. Maputo	601 (14.5)	148 (11.9)	749 (13.9)	1.3 (0.62–719)	0.62		
	Other	18 (0,4)	3 (0.2)	21 (0.4)	1.5 (0.43–5.08	0.53		
Follow-up time (years)	2–5	523 (12.6)	282 (22.6)	805 (14.9)	Ref			
	6–9	637 (15.4)	125 (10.0)	762 (14.1)	0.4 (0.29–0.46)	< 0.001	0.7 (0.48–1.07)	0.10
	10 +	2988 (72.0)	840 (67.4)	3828 (71.0)	0.5 (0.47–0.62)	< 0.001	1.1 (0.77–1.43)	0.75
Current ART regimen^a^	TDF + 3TC + DTG	3824 (92.2)	418 (33.6)	4242 (78.7)	Ref			
	TDF + 3TC + EFV	191 (4.6)	293 (23.6)	484 (9.0)	14.0 (11.39–17.29)	< 0.001	12.7 (9.74–16.63)	< 0.001
	AZT + 3TC + NVP	42 (1.0)	154 (12.4)	196 (3.6)	33.5 (23.50–47.88)	< 0.001	21.8 (14.13–33.59)	< 0.001
	Other	91 (2.2)	378 (30.4)	469 (8.7)	38.0 (29.59–48.80)	< 0.001	25.8 (18.58–35.80)	< 0.001
Type of patient^a^	Military	479 (11.8)	206 (18.6)	685 (13.2)	1.7 (1.44–2.05)	< 0.001	1.1 (0.86–1.44)	0.40
	Civil	3592 (88.2)	900 (81.4)	4492 (86.8)	Ref			
Kaposi's sarcoma	Yes	4009 (96.6)	1231 (98.7)	5240 (97.1)	2.7 (1.58–4.49)	< 0.001	1.2 (0.65–2.23)	0.56
	No	139 (3.4)	16 (1,3)	155 (2.9)	Ref			
Active tuberculosis	Yes	130 (3.1)	59 (4.7)	189 (3.5)	1.5 (1.12–2.10)	0.008	4.6 (3.15–6.63)	< 0.001
	No	4018 (96.9)	1188 (95.3)	5206 (96.5)	Ref			
WHO Stadium^a^	SI	393 (9.5)	140 (11.2)	533 (9.9)	-			
	I	1092 (26.3)	365 (29.3)	1457 (27.0)	1.2 (1.02–1.42)	0.03	1.0 (0.79–1.29)	0.94
	II	965 (23.3)	276 (22.1)	1241 (23.0)	1.0 (0.87–1.23)	0.72	1.0 (0.79–1.27)	0.97
	III	1376 (33.2)	381 (30.6)	1757 (32.6)	Ref			
	IV	322 (7.8)	85 (6.8)	407 (7.5)	1.0 (0.73–1.24)	0.72	1.1 (0.78–1.56)	0.59
Outcome	Active	3920 (94.5)	1097 (88.0)	5017 (93.0)	Ref			
	Abandonment	37 (0.9)	42 (3.4)	79 (1.5)	4.1 (2.59–6.34)	< 0.001	–-	–-
	Death	51 (1.2)	54 (4.3)	105 (1.9)	3.8 (2.57–5.58)	< 0.001	–-	–-
	Transferred	140 (3.4)	54 (4.3)	194 (3.6)	1.4 (0.99–1.90)	0.05	–-	–-

***Where****: WHO-World Health Organization, SI-no information, ART-antiretroviral treatment, OR-odds ratio, AOR-adjusted odds ratio, TDF-tenoforvirdisoproxifumarate, 3TC-lamivudine, DTG- dolutegravir, EFV-enfaverence, AZT-azidothymidine, NVP-nevirapine*

^*a*^*Variable where the N was less than n* = *5395 due to unfilled fields *Table 3

In the multivariate analysis, after adjusting for potential confounding variables, several factors remained significantly associated with viral non-suppression. Single marital status had a significantly higher likelihood of viral non-suppression compared to those in a marital union, with an adjusted odds ratio (AOR) of 4.8 (95% CI: 3.93–5.76, *p* < 0.001). The ART regimen also proved to be statistically significant. Specifically, the TDF + 3TC + EFV regimen had an AOR of 12.7 (95% CI: 9.74–16.63, *p* < 0.001), the AZT + 3TC + NVP regimen had an AOR of 21.8 (95% CI: 14.13–33.59, *p* < 0.001), and other regimens had an AOR of 25.8 (95% CI: 18.58–35.8, *p* < 0.001) when compared to the TDF + 3TC + DTG regimen. Additionally, having been diagnosed with active TB was significantly associated with viral non-suppression compared to not having been diagnosed with TB, with an AOR of 4.6 (95% CI: 3.15–6.63, *p* < 0.001) (Table 3).

## Discussion

This study analyzed retrospective data from military and civilian patients on ART at CITRA/HMM with the aim of estimating the proportion of patients with viral suppression and identifying the factors associated with non-virological suppression. Our analysis revealed minimal differences in gender distribution among the patients studied. Despite the Mozambique Armed Defense Forces (FADM) predominantly comprising males, there was a marginally higher representation of female patients in our study. CITRA operates maternal and child health services, and all women who seek these services must undergo an HIV test. If diagnosed as HIV positive, they are enrolled in ART services. Additionally, it is scientifically proven that women tend to take better care of their health, which drives them to seek healthcare services more often than men [12–14].

Viral suppression was achieved by only 76.9% of patients, which is below the UNAIDS target (third 95%) [2]. Recent data from INSIDA 2021 highlighted that the overall viral suppression rate among patients on ART in Mozambique reached 89.4%. Notably, in Maputo province and Maputo City, where the majority of our study's cases were located, patients on ART exhibited even higher rates of viral suppression, with 91.1% in Maputo province and 92.5% in Maputo City, respectively [3]. Military personnel exhibited lower viral suppression rates, although the differences were not statistically significant after adjustment, aligning with findings from other studies, that indicates that military personnel may require intensive ART adherence counseling to achieve optimal suppression [15–19].

The study identified single marital status, specific ART regimens, and active TB diagnosis as factors associated with non-achievement of viral suppression. Dolutegravir-based ART (DTG) regimens demonstrated better outcomes, affirming recent research advocating for DTG's efficacy. However, achieving viral suppression remains complex, influenced by a myriad of factors, including socio-demographic and genetic variations, and the prevalence of HIV/TB co-infection, particularly in African settings [20] [21].

The correlation our study identified between active TB and HIV is a prevalent issue in many African countries [16, 22–24] and is known to obstruct to viral suppression. This coinfection is a major public health threat, responsible for an alarming number of deaths worldwide [25]. These figures reflect a synergistic effect between the deleterious outcomes of both infections, creating a concept referred to as a “synergistic epidemic” where HIV and TB mutually amplify each other’s pathogenic potential. In this interaction, the macrophages take up the pathogen, but TB impairs phagolysosome fusion and autophagy. This is enhanced during HIV co‐infection, which helps TB to establish intracellular niches. In turn, TB increases the expression of the HIV coreceptors CXCR4 and CCR5 (helping HIV spread during coinfection) [26].

A study from Uganda has demonstrated the “synergistic epidemic” (HIV and TB) where that the combination of low adherence to ART and active TB significantly elevated the risk of failing to achieve viral suppression [22]. Furthermore, a 2021 study from the Democratic Republic of Congo highlighted the serious implications of HIV/TB co-infection, increasing not only the likelihood of viral non-suppression but also the risk of mortality among patients [24]. In this study it was not possible to accurately assess adherence because of the lack of data on absenteeism, which is a crucial factor in measuring individual adherence. In addition, the available data on medication adherence were limited. Although patients who discontinued treatment, had been transferred or died initially appeared to be associated with non-viral suppression, these associations lost statistical significance after adjustment. However, previous research emphasises the critical role of adherence to ART in boosting immunity and reducing viral replication [27, 28].

Research into individual factors associated with non-adherence to ART revealed that non-participation in a support group for PLHIV was associated with an increased risk (43%) [29], although participation rates were low [29, 30]. The military setting, characterized by its insularity, may exacerbate the challenges of maintaining confidentiality and amplify stigma, potentially affecting participation in support groups.

This study had several limitations that impacted the findings. One of the major constraints was data quality, exemplified by only 46% of participants in the FADM HIV Program in 2018 having a documented viral load [6], which reflects broader issues in data integrity. These limitations affected sample sizes across various analysis sections, potentially skewing the findings and diminishing the robustness of the results. Additionally, the lack of comprehensive data on absenteeism and medication adherence further hindered accurate assessment of adherence levels. Another limitation is the timing of the analysis, which concluded in 2020, potentially not fully capturing the impact of recent HIV Program updates. Furthermore, the analysis did not account for the potential impacts of the COVID-19 pandemic, which has significantly affected healthcare access and ART adherence globally. Consequently, the results' applicability may vary based on local service availability and recent program changes. These limitations should be considered when interpreting the study's findings, highlighting the need for improved data quality and ongoing monitoring to better understand and address the factors influencing viral suppression.

## Conclusion

This study sheds light on the multifaceted challenges in achieving viral suppression among military and civilian populations undergoing ART in Mozambique. While socio-demographic factors, specific ART regimens, and co-infections like TB play significant roles, the underlying issues of ART adherence, data quality, and the unique challenges within the military setting further complicate the HIV treatment landscape. These findings underscore the need for targeted interventions, improved data management, and a comprehensive approach to addressing the barriers to viral suppression, particularly in populations with distinct needs such as military personnel. As Mozambique continues to fight the HIV epidemic, these insights are crucial for shaping strategies that are sensitive to the nuances of the affected populations and the complexities of the disease.

## Data Availability

The data supporting the conclusions of this study are available at the Maputo Military Hospital, but restrictions apply to the availability of these data, which were used under license for the current study and are therefore not publicly available. However, the data may be made available by the authors upon reasonable request and with permission from the National Directorate of Military Health and the Department of Military Health of General Staff of Mozambique. contact: otuzine@yahoo.com.br .

## Abbreviations

3TC: Lamivudine
AIDS: Acquired Immune Deficiency Syndrome
AOR: Adjusted Odds Ratio; ART: Antiretroviral therapy
AZT: Azidothymidine
CD-4: Cluster of Differentiation 4
CDC: Centers for Disease Control and Prevention
CI: Confidence Interval
CIBS FM&HCM: Institutional Health Bioethics Committee of the Faculty of Medicine/Central Hospital of Maputo
CITRA: Integrated Treatment Center
COR: Crude Odds Ratio
DTG: Dolutegravir
FADM: Mozambique Armed Defense Forces
EFV: Efavirenz
HIV: Human Immunodeficiency Virus
IQR: Interquartile range
MMH: Maputo Military Hospital
NVP: Nevirapine
PEPFAR: US President’s Emergency Plan For AIDS Relief
PLHIV: People Living With HIV/AIDS
P-value: Precession value
Ref: Reference
TB: Tuberculosis
TDF: TenofovirDisoproxilFumarate
UNAIDS: Joint United Nations Programme on HIV and AIDS
VL: Viral Load
WHO: World Health Organization
